# A Novel Electroactive Agarose-Aniline Pentamer Platform as a Potential Candidate for Neural Tissue Engineering

**DOI:** 10.1038/s41598-017-17486-9

**Published:** 2017-12-07

**Authors:** Payam Zarrintaj, Behnaz Bakhshandeh, Iraj Rezaeian, Behnam Heshmatian, Mohammad Reza Ganjali

**Affiliations:** 1 School of Chemical Engineering, College of Engineering, University of Tehran, Tehran, Iran; 2 Department of Biotechnology, College of Science, University of Tehran, Tehran, Iran; 3 Neurophysiology Research Center, Urmia University of Medical Sciences, Urmia, Iran; 4 Center of Excellence in Electrochemistry, School of Chemistry, College of Science, University of Tehran, Tehran, Iran; 5 Biosensor Research Center, Endocrinology and Metabolism Molecular-Cellular Sciences Institute, Tehran University of Medical Sciences, Tehran, Iran

## Abstract

Neuronal disorder is an important health challenge due to inadequate natural regeneration, which has been responded by tissue engineering, particularly with conductive materials. A bifunctional electroactive scaffold having agarose biodegradable and aniline pentamer (AP) conductive parts was designed that exhibits appropriate cell attachment/compatibility, as detected by PC12 cell seeding. The developed carboxyl-capped aniline-pentamer improved agarose cell adhesion potential, also the conductivity of scaffold was in the order 10^−5^ S/cm reported for cell membrane. Electrochemical impedance spectroscopy was applied to plot the Nyquist graph and subsequent construction of the equivalent circuit model based on the neural model, exhibiting an appropriate cell signaling and an acceptable consistency between the components of the scaffold model with neural cell model. The ionic conductivity was also measured; exhibiting an enhanced ionic conductivity, but lower activation energy upon a temperature rise. Swelling behavior of the sample was measured and compared with pristine agarose; so that aniline oligomer due to its hydrophobic nature decreased water uptake. Dexamethasone release from the developed electroactive scaffold was assessed through voltage-responsive method. Proper voltage-dependent drug release could be rationally expected because of controllable action and elimination of chemically responsive materials. Altogether, these characteristics recommended the agarose/AP biopolymer for neural tissue engineering.

## Introduction

Human organ loss occurs usually due to lethal events such as accident or disease. Due to complexity of natural neural healing and prevalence of neural disorders, development of the novel and effective therapeutic approaches has far been addressed by many researches^1–3^. Tissue engineering scrambles to compensate for the tribulation resulting from organ loss. Appropriate cell source, effective cell modification and proper supportive matrices are main bases of tissue regeneration^4–6^. Polymers play indispensable roles as supportive matrices in tissue engineering and subordinate of regenerative medicine. There are two types of polymers that have been used in different biomedical applications: (i) the first types are synthetic polymers like poly lactic acid (PLA)^7^ and polycaprolactone (PCL)^8^, and the second types are (ii) natural polymers such as agarose^9^, chitosan^10^ and gelatin^11^.

Other parameters like growth factors^12^, drugs^13^, scaffold topology and biochemistry^14^ may also affect the seeded-cell differentiation and adhesion. As the cell proliferation can pose negative effect on cell membrane potential, conductive scaffolds can resolve such situation by electrical communication among cells and intracellular activities^15^. The appropriate conductivity for intracellular activity was proposed to be in between 10^−7^–10^−2^ S/cm, depending on tissue. In particular in neural and cardiac tissue engineering^16^, due to their nature, the electrical activity is more salient. The data transfer in neural cells occurs by action potential phenomenon stimulating with conductive substrate. It was demonstrated that the scaffold conductivity is more important than that of modulus for cell proliferation^17,18^.

There are a lot of different types of materials such as carbon nanotube (CNT)^19^, graphene^20^, graphene oxide^4^ and conductive polymers^21^, which have been utilized for electrical conductivity amplification. Nonetheless, these types of materials have some drawbacks such as non-biodegradability^22^, poor solubility^23^ and chronic inflammation owing to their long lifetime in the body^24^. In order to deal with these kind of problems, there are some new electroactive biopolymers have been utilized based on conductive oligomers such as aniline oligomers^18^. Generally, electroactive biocompatible polymers based on oligomers have the oligo conductive part and biodegradable part which are responsible for conductivity and bio-compatibility, respectively^25^.

Oligoaniline is a kind of proper conductive materials that are used to produce electroactive biopolymer because of its affluence and easiness of synthesis. Accordingly, it can be employed in drug delivery^26^, nerve probe^27^, biosensor^28^, neural^18^ and cardiac^29^ scaffolds. Oligoaniline can be consumed by macrophage and purified with kidney; As such, there isn’t any evidence of harmful side-effect in the body^30–32^ while having sufficient conductivity for human tissues^33–35^.

Neural recording is one of the challenging issues in tissue engineering. At cellular level, neural electrodes are interface with central and peripheral nerves with assisting the biological signal transduction to electronic one^36^. Minimizing the impedance electrode/tissue interface is the significant factor for appropriate signaling. To this aim Abidian *et al*. used poly(3,4-ethylenedioxythiophene) (PEDOT) nanofibers as a coating for electrodes. Dexamethasone was used as an anti-inflammatory drug to reduce the trauma and inflammation to keep the impedance in low level. Moreover alginate based hydrogel was coated the electrode to adjust the electrode modulus with soft tissue and controlled the release^37^.

**Figure 1:**
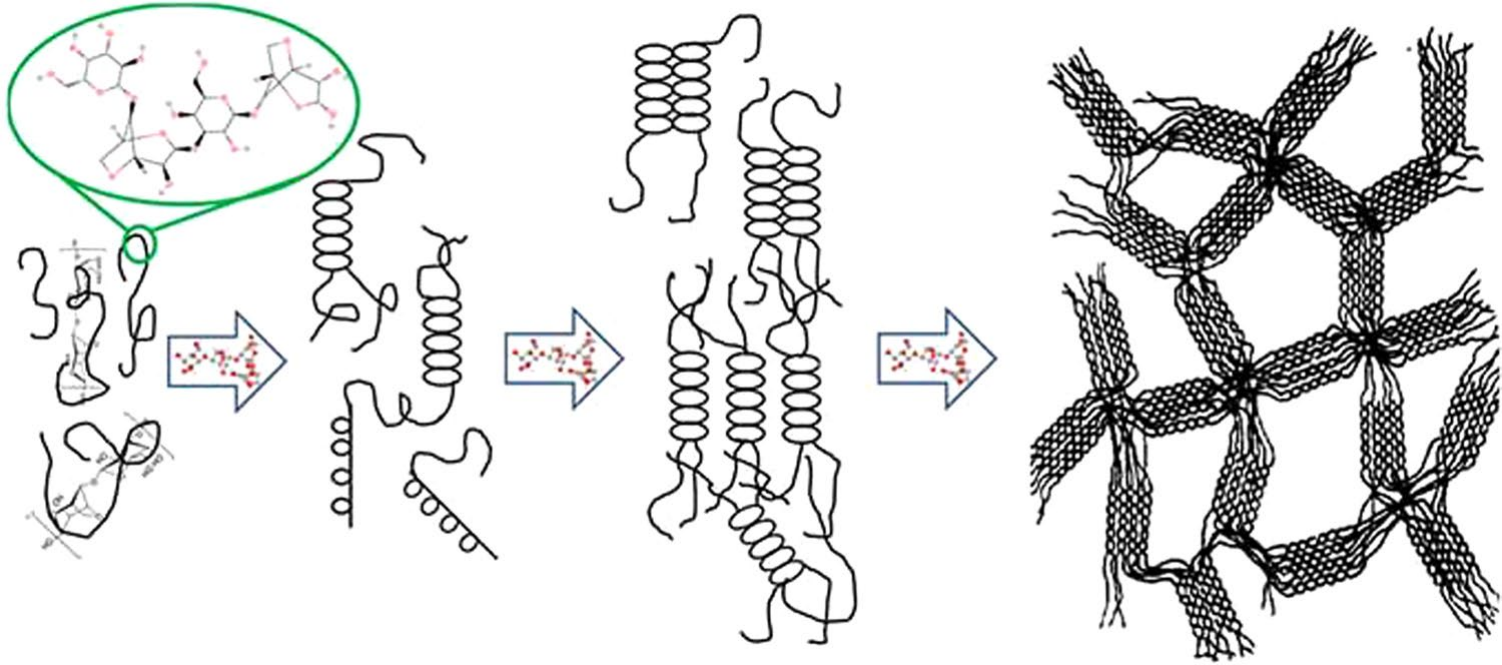
Gelation Mechanism of Agarose: agarose chains due to the hydrogen bonds and electrostatic interaction tend to form helical structure and gel.

One of the challenging issues in hydrogel formation is gelation behavior. Crosslinking agents form 3D structure of hydrogel and endow the mechanical durability; however, they are usually toxic and exhibit deleterious effect on cells. Self-gelling hydrogels like agarose do not need crosslinking agent and form the gel solitarily because of hydrogen bond, electrostatic interaction and helical structure formation (Fig. 1)^38^. Self-gelling material like agarose can be used with other polysaccharides like alginate to reduce the cross-linking agent usages.

In our previous work, alginate-tetramer was synthesized and agarose was used for forming a hydrogel because of its inert and non-immunological features^39^. It was hypothesized that the aniline oligomers presence in alginate structure enhanced the conductivity. Ionic conductivity increased and the activation energy decreased with temperature increment because of the ions mobility enhancement. Moreover, agarose was utilized to obviate the toxic effect of crosslinking agent along with enhancing the mechanical features^39^. However, it is assumed that the agarose presence in system decrease the ionic conductivity because of large size of molecule which act as a barricade between conductive units.

During this study, we synthesized agarose-coupled aniline pentamer scaffold as a novel electro-active biopolymer. Agarose is a biocompatible polymer and aniline pentamer has enough conductivity when doped with camphor sulfonic acid. Various elements such as electrical activity, ionic conductivity, impedance spectroscopy, topology, thermal properties, cytotoxicity and biocompatibility were inspected to characterize the scaffolds for neural tissue engineering applications.

## Result

### Scaffold Characterization

**Figure 2:**
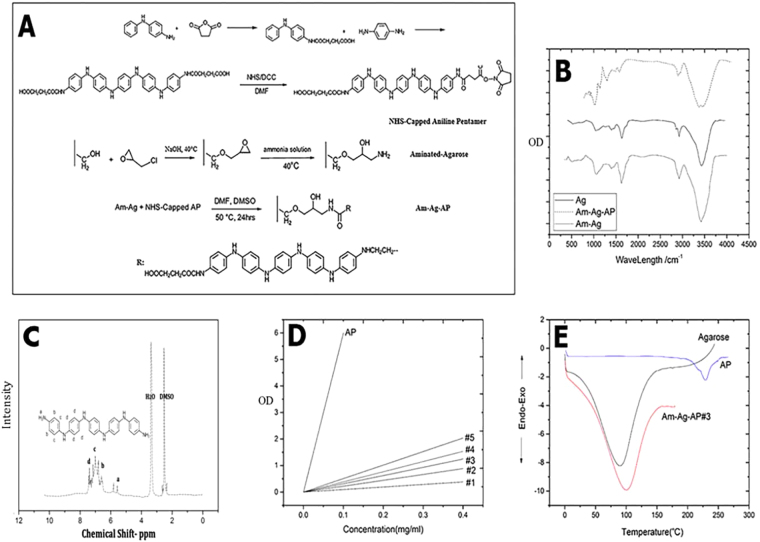
Scaffold synthesis and characterization; (**A**) A scheme of Am-Ag-AP synthesis, (**B**) FTIR spectra of agarose, aminated agarose and Am-Ag-AP, (**C**) HNMR spectra of Aniline Pentamer, (**D**) AP concentration in samples in comparison to the Pure AP; (**E**) Differential scanning calorimetry (DSC): coupling AP into agarose resulted in melting point increment.

Aminated agarose-aniline pentamer (Am-Ag-AP) synthesis method is depicted in Fig. 2A. Firstly, NHS-capped aniline pentamer was synthesized; then, it was coupled with the aminated agarose to get aniline pentamer-agarose. FTIR peaks are exhibited in Fig. 2B for aniline pentamer (AP), aminated agarose (Am-Ag) and Am-Ag-AP. For measuring agarose amination degree, TNBS assay was conducted showing 48%. H-NMR test was carried out for AP characterization (Fig. 2C). Moreover, the AP concentrations in samples were determined by UV-Visible; Fig. 2D shows relevant concentration.

### Electro Activity and Conductivity Evaluation

**Figure 3:**
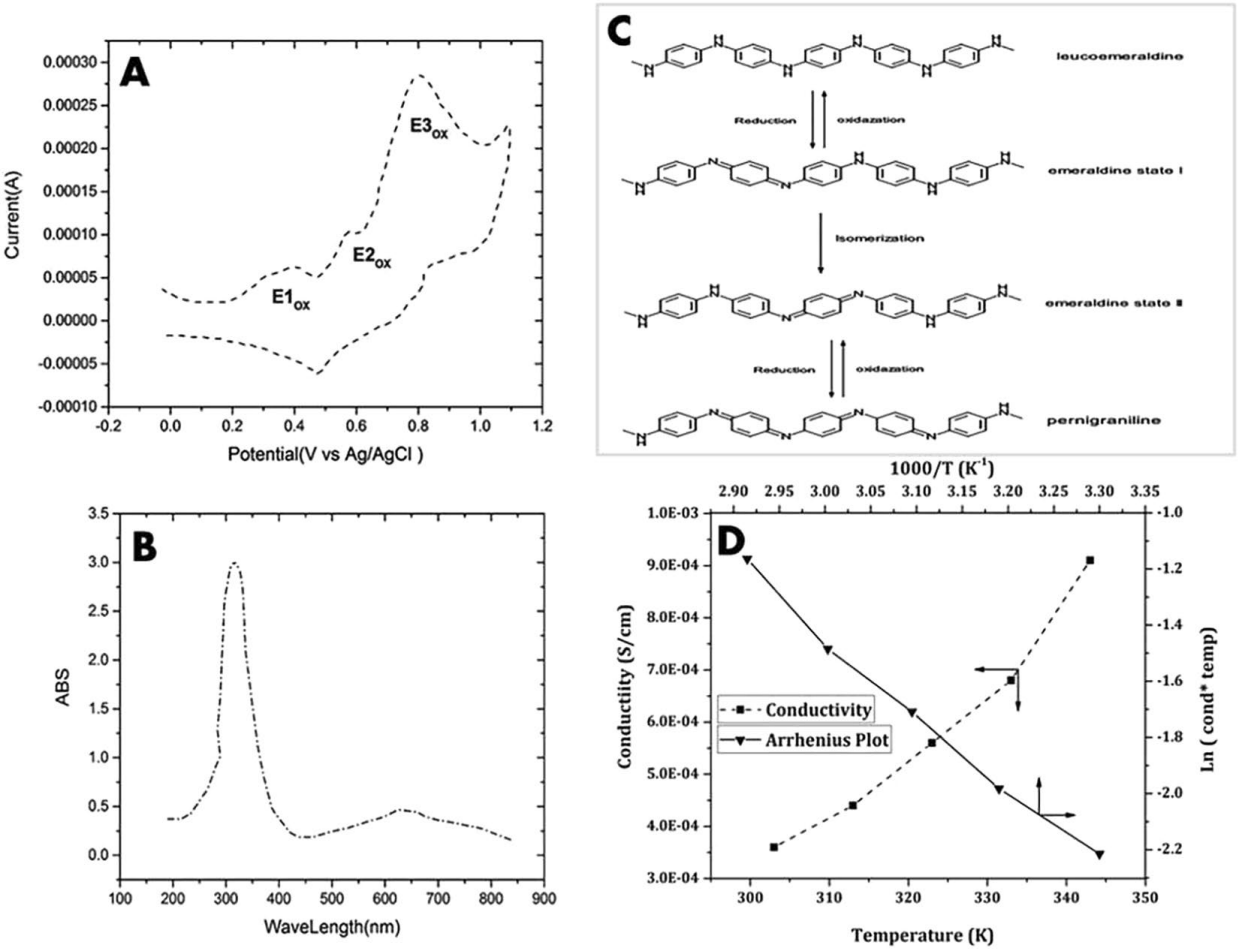
Electrochemical analyses of the scaffolds; (**A**) Cyclic Voltammetry of Am-Ag-AP: the sample electroactivity is featured by three oxidation/reduction peeks, (**B**) UV-V spectrum of aniline pentamer: two transition peaks related to π-π^*^ and π_b_-π_q_ are obvious, (**C**) The scheme of Aniline Pentamer transition in oxidation-reduction activities from oxide to reduced states, (**D**) The ionic conductivity of Am-Ag-AP.  Doping AP via camphor sulfonic acid caused emeraldine state and provided proper conductivity.

Cyclic voltammetry measurement is depicted in Fig. 3A. Three oxidation with reversible peaks are observed in 0.41, 0.58 and 0.79 V (E1/2 = (Epa + Epc)/2) indicating AP shuttles among these states (Fig. 3C). Furthermore, the UV spectrometry displays two peaks in Fig. 3B. Moreover, scaffold conductivity in ultimate swollen state was evaluated. The conductivity increment with temperature enhancement is depicted in Fig. 3D; moreover, the activation energy was 22.44 and 15.7 for 30 and 70 °C, respectively. The highest ionic conductivity was around 9.1 × 10^−4^.

**Figure 4:**
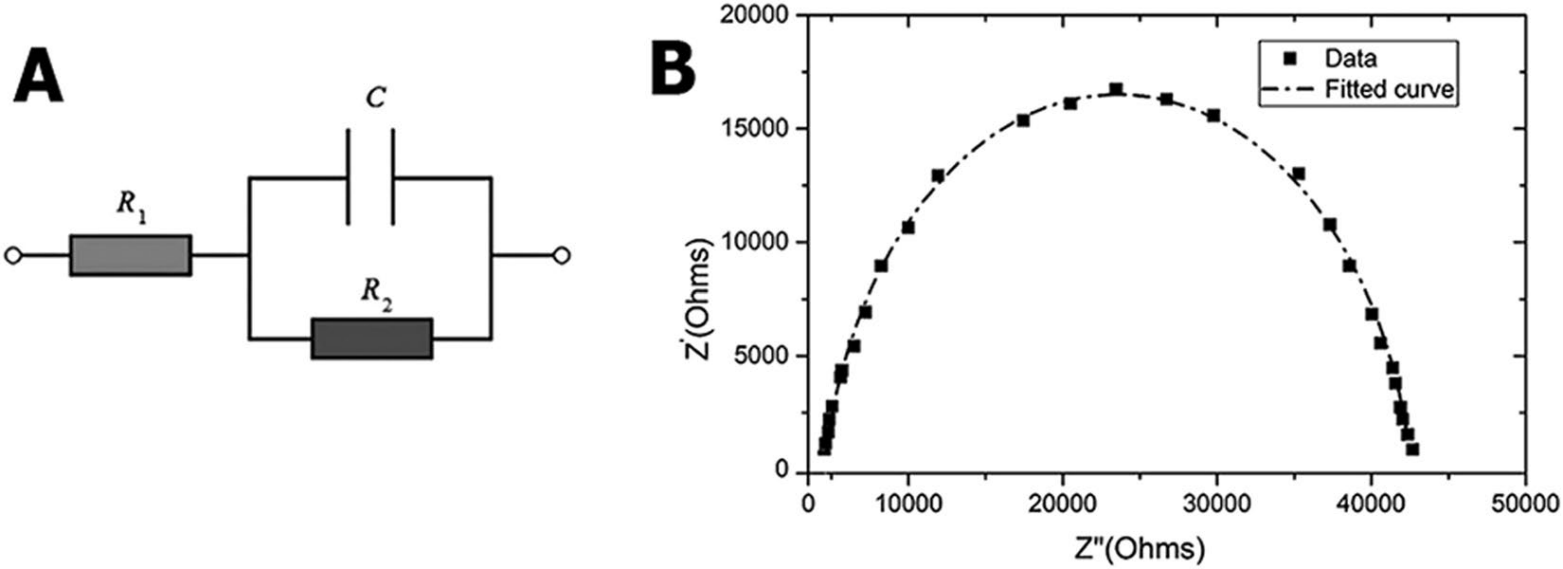
Electrical conductivity evaluation; (**A**) Equivalent circuit model for neural induction, (**B**) Nyquist plot of Am-Ag-AP which was yielded from electrochemical impedance spectroscopy that it was used for gaining the circuit model components.

For calculating the equivalent circuit model electrochemical impedance spectroscopy was done by using the neural model, this model was consisted of two resistors and one capacitance demonstrated in Fig. 4A. With EIS data, Nyquist plot was plotted which is shown in Fig. 4B. Through using this model, EIS data was expounded and also the model elements were calculated. C, R_1_ and R_2_ were around 1.318 μF/cm^2^, 1986.3 mΩ*cm^2^ and 38680 3 mΩ*cm^2^, respectively, all of which were calculated using EIS spectrum analyzer software.

### Physical Properties: Thermal and Morphology

**Figure 5:**
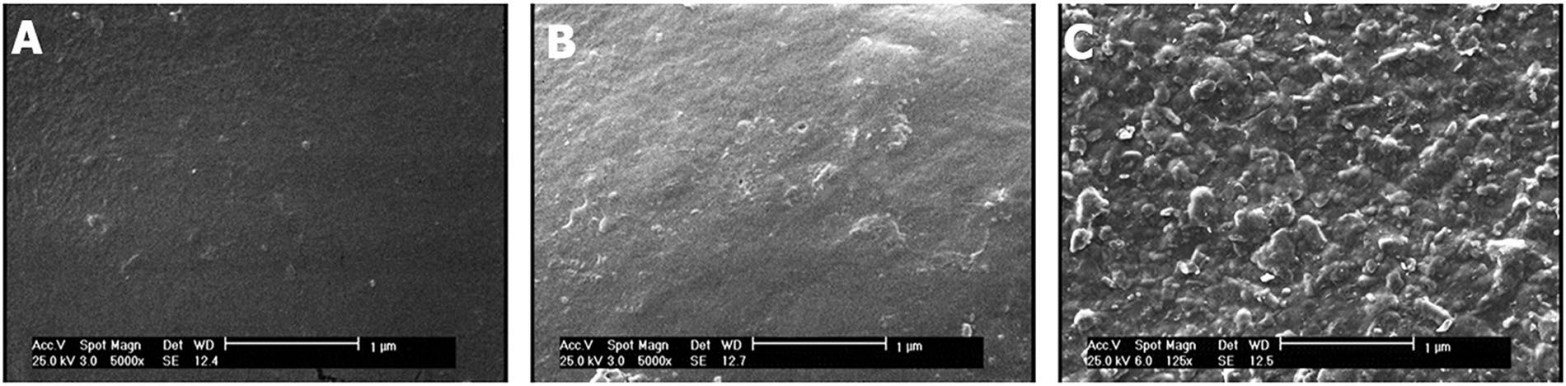
SEM analysis of the scaffolds; (**A**) pure agarose, (**B**) Am-Ag-AP#3, (**C**) Am-Ag-AP#5, addition of hydrophobic AP caused self-assembled spheres formation.

Some of the thermal attributes of the samples were persuaded with differentiation scanning calorimetry (DSC). The pure agarose, AP and Am-Ag-AP#3 exhibited melting point around 90 °C, 210 °C and 100 °C, respectively. DSC curve of samples are shown in Fig. 2E. Also, the morphology of samples is shown in Fig. 5. The Vesicles organized by elevating AP content because of amphiphilic properties of Am-Ag-AP.

### Swelling and Drug Release Evaluation

**Figure 6:**
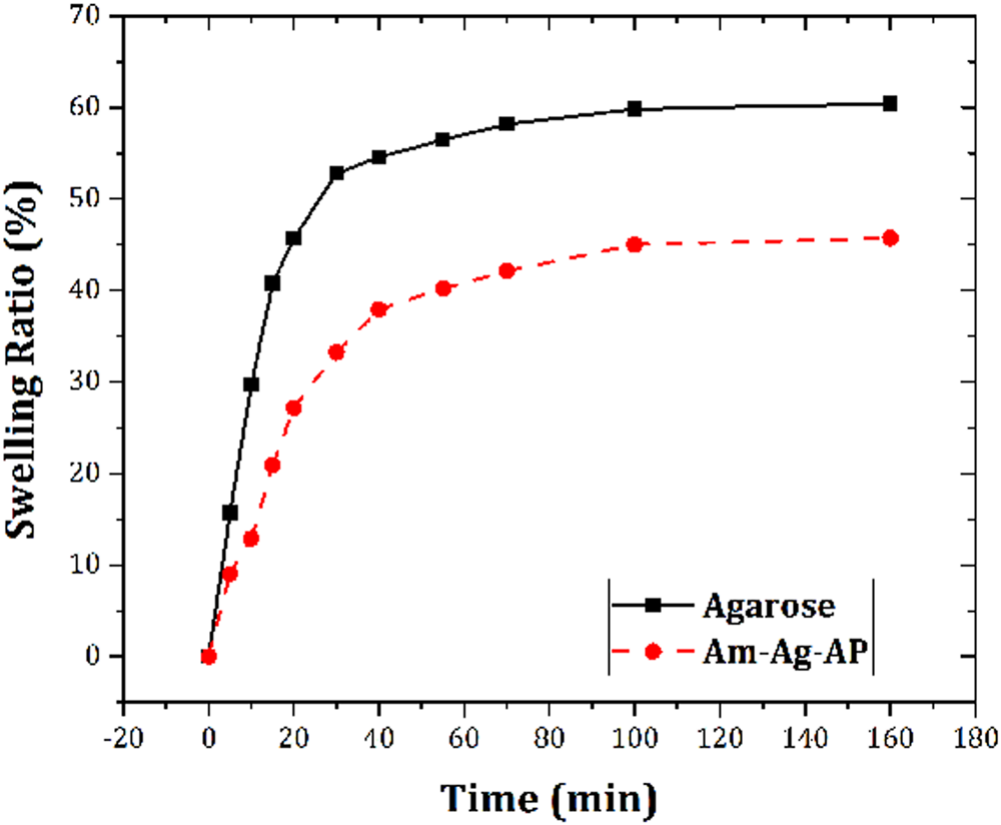
Swelling evaluation of Am-Ag-AP and Pristine Agarose: aniline pentamer resulted in water-uptake decrement because of its hydrophobic nature.

Swelling profile is depicted in Fig. 6 for pristine agarose and Am-Ag-AP. The results revealed that the aniline pentamer coupling to agarose decreased the swelling ratio.

**Figure 7:**
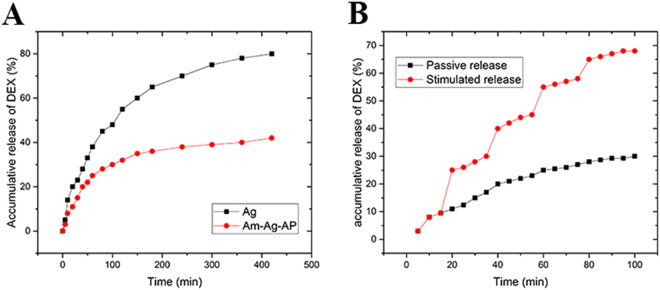
Accumulative release of Dexamethasone; (**A**) passive release (**B**) stimulated release: the conductive sample showed the controlled release according to the electrical stimulation.

Passive and stimulated release of samples was evaluated, and the resulting patterns are shown in Fig. 7. The released amount of drug was registered at various time points and the accumulative drug release profile was drawn. It is obvious that passive drug release from agarose was more than Am-Ag-AP, while drug release quantity and the release rate were increased by electrical stimulation.

### Biocompatibility and Cytotoxicity Analyses

**Figure 8:**
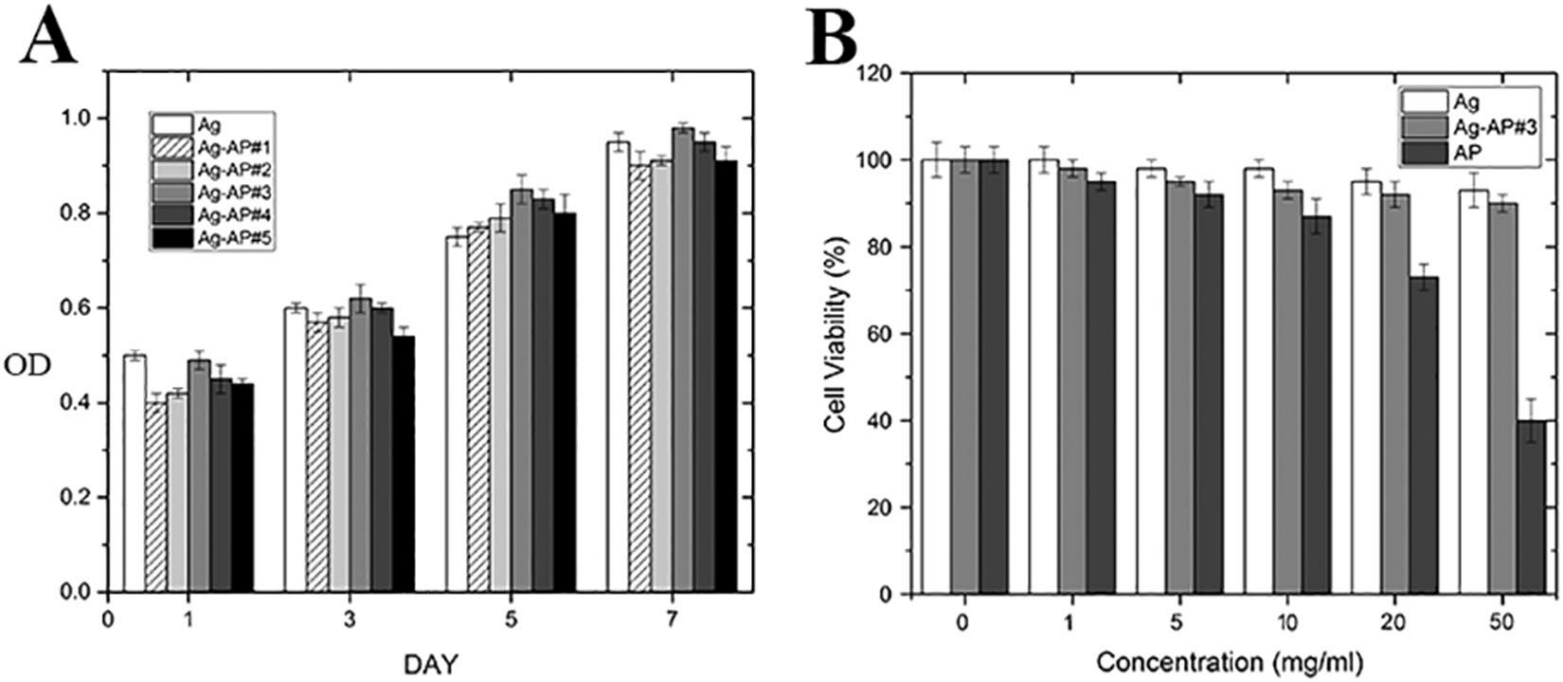
Biocompatibility evaluations; (**A**) by MTT assay of all scaffolds, (**B**) by cell proliferation analysis of the Am-Ag-AP#3. Sample #3 revealed proper features for biological applications such as tissue engineering.

Biocompatibility and cytotoxicity analysis were also investigated. The samples showed a proper compatibility with cells, this compatibility was especially observable in sample number 3 (Am-Ag-AP#3) which exhibited the best properties among other samples and pure agarose (Fig. 8A). Am-Ag-AP#3 that exhibited the best cell proliferation was evaluated for cytotoxicity. For this purpose, different concentrations of secreted liquid from Am-Ag-AP#3 were subjected to PC12 cells to determine its cytotoxicity. The results are shown in Fig. 8B illustrating that the Am-Ag-AP#3 had no cytotoxicity.

**Figure 9:**
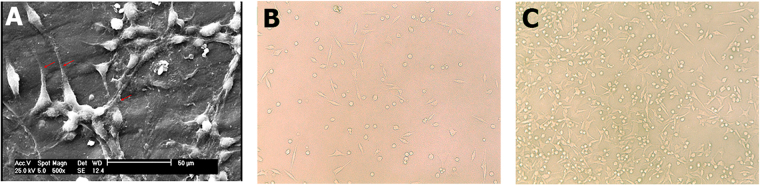
Cell adhesion and proliferation: (**A**) SEM image of seeded PC12 cells on sample Am-Ag-AP#3; the adherence of PC12 cells is obvious in result of the scaffold biocompatible properties, (**B**) phase-contrast microscopy of PC12 proliferation in the medium containing 50 mg/ml concentration of AP, (**C**) phase-contrast microscopy of PC12 proliferation in the medium containing 50 mg/ml concentration of Am-Ag-AP#3. The results indicated that the sample#3 excretion medium has no significant cytotoxic effect and suggests it for *in-vivo* applications. 100X magnification.

The SEM image of PC12 seeded on the Am-Ag-AP#3 is shown in Fig. 9A which shows the adhesion of cells to scaffold; also, optical microscopy images (Fig. 9B,C) were captured for cytotoxicity results concerning the cell proliferation with AP and Am-Ag-AP#3 excreted liquid showing that the AP caused lower cell proliferation; on the contrary, Am-Ag-AP#3 caused cell have enough proliferation.

## Discussion

Polysaccharides are biomedical, biocompatible and degradable polymers that exhibit satisfactory results in nerve regeneration because of axonal out growth^40,41^. Yet, these types of scaffold could not regenerate the defect in large breaks between distal and proximal in peripheral nerve injury; thus, stimulators should be used in scaffold layout. For achieving better nerve repair, conductivity motivates neurite consequence, axonal regeneration and cellular activity^18,42,43^. Moreover, employing scaffolds in which cells and growth factors are embedded such as stem cell^9^, Schwan cell^18^ and neurotropic factor^12^ can promote the neural morphogenesis. It is noteworthy mentioning that in neural prosthesis fabrication, high charge density, low impedance and soft modulus electrode are the outcomes considered for neural recording and stimulating^37^.

In this project, we tried to synthesis a bio conductive polymer in order to have biocompatibility and electrical conductivity. AP was coupled with agarose that the reaction is depicted in Fig. 2A. In a word, NHS and DCC (i.e. carbodiimides play the role as a coupling agent. NHS was used to gain NHS-capped as a dry-product which had an enough stability) were used to link carboxyl capped aniline pentamer to aminated agarose. This method was used to couple the aniline pentamer with polysaccharides which have a pendant amine group such as chitosan^44^ and gelatin^45^.

As it is exhibited in Fig. 2B, Agarose FTIR peaks were around 3550 cm^−1^ related to O-H stretching bond, 2890 cm^−1^ related to CH_2_, 1655 cm^−1^ related to H-O-H water stretching vibration bond to polysaccharides, 1034 cm^−1^ assigned to glycoside stretching bond and 929 related to C-O-C stretching vibration of 3,6 anhydro-L-glactopyrose. Agarose aminated peaks are 3560 and 3472 cm^−1^ related to –NH_2_ stretching bond, 1593 and 1245 cm^−1^ related to N-H bending vibration and C-N stretching vibration that proves the ring opening of the epoxy ring with ammonia^46^. Am-Ag-AP peaks were around 1490 and 1570 cm^−1^ due to C=C benzoid (-N-B-N) and quinoid (-N=Q=N-) ring in AP also 1300 and 1230 cm^−1^ related to C-N stretching benzoid and quinoid ring^47^.

Figure 3A in which the cyclic voltammetry is illustrated, exhibits three oxidations with reversible peaks that refer to the transition from leucoemeraldine to emeraldine state Ι, emeraldine state Ι to emeraldine state ΙΙ and emeraldine state ΙΙ to pernigraniline state, respectively. Molecular resonance is depicted in Fig. 3C.

**Figure 10:**
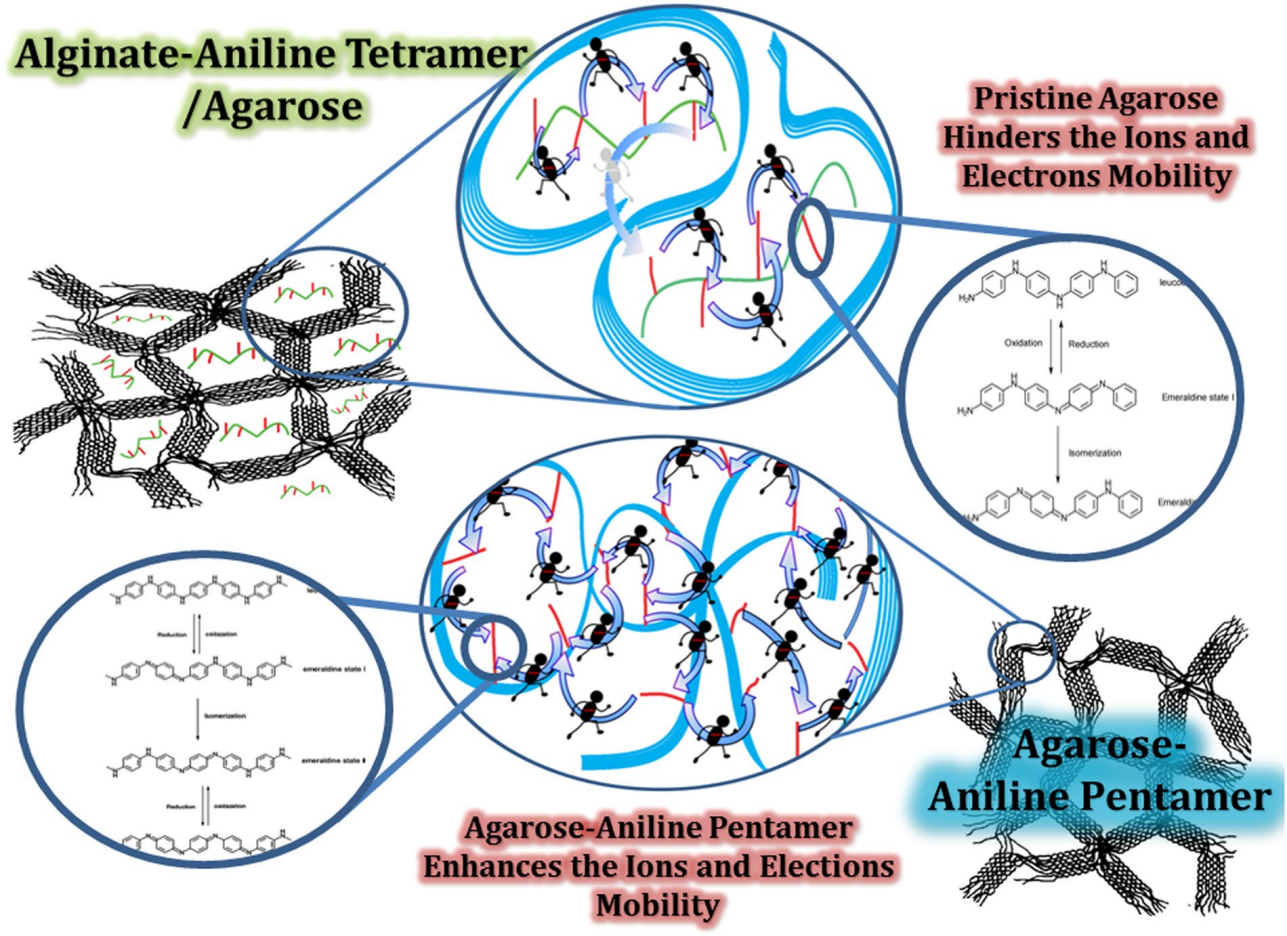
Comparison of alginate-aniline tetramer and agarose-aniline pentamer, ionic conductivity. Aniline tetramer exhibit two redox states but aniline pentamer exhibit three oxidation peak which cause to better ionic mobility. In alginate-aniline tetramer, agarose just act as a gelling agent and decreases the connection of alginate-aniline tetramer segments, on the other hand, in Am-Ag-AP, aniline pentamers are located on the agarose chain and there is no hindrance among conductive units; hence agarose-aniline pentamer acts better than alginate-tetramer.

Pursuant to Fig. 3B, it is obvious that UV-Vis spectroscopy shows two peaks around 320 and 600 nm that is related to the π-π^*^ transition of the benzene ring and the π_B-_π_Q_ transition (benzenoid to quinoid excitonic transition), respectively. Moreover, with EIS data, as it is explained in Fig. 4B Nyquist plot was plotted. A bio conductive polymer shows a supreme attraction to be employed as a neural and cardiac scaffold because of their conductive properties. Owing to action potential properties of the neural system it was supposed to have various equivalent circuit models were. The basic neuron model includes two resistances and one capacitor. Due to neuron membrane charge storage and separation ability,it plays the capacitor role; furthermore, ion channels because of their ion mobility, play the role of the conductance. As a passive transport, ion channel was shown with resistance in which more ions able to flow, it will be decreased^48^. The calculated capacitance from EIS data was close to the different types of the neural cell like glial cell, hippocampal neuron, spinal cord neuron and cortical pyramidal neuron whose capacitance were around 0.9–1.1 μF/cm^2,49^. As discussed before, hydrogel-based material was used as a coating for neural electrodes to regulate the electrode modulus with soft tissue modulus. It was reported that the drug loaded alginate could be considered for electrode coating to adjust the modulus. However, the insufficient conductivity of the alginate may cause deterioration of conductivity and impedance ending in a fall in the neural electrode efficiency^37^. Moreover, for alginate hydrogel preparation, a crosslinking agent like CaCl_2_ is needed, which may unavoidably lead to the electrode corrosion with negative effect on cells. From this perspective, agarose-aniline pentamer can be used as a coating for electrodes to tune the modulus of the electrode without impedance enhancement, in turn with positive effects on the neural electrode capability. Moreover, the absence of toxic and corrosive crosslinking agents in agarose-aniline pentamer platform in this work improved self-gelling properties; hence, the cell viability could be expected to ameliorate with less corrosion. It was supposed that, scaffold electrochemical features proximity to cells resulted in better cell proliferation and growth. Because of cells membrane potential, conductive scaffolds have more compatibility with cells. To achieve desire conductivity samples should be doped with a proper dopant, camphor sulfonic acid was used as a dopant because it showed a suitable compatibility with the cell^29^. As it is shown in Fig. 3D, ionic conductivity enhanced and activation energy declined with temperature increment because of higher mobility of ions at higher temperature. In comparison with alginate-aniline tetramer/agarose systems^39^, it was observed that the activation energy of agarose-aniline pentamer was lower and ionic conductivity was higher. It was assumed that the aniline oligomer presence in matrix enhanced the ionic conductivity^39^. As regards to the alginate-aniline tetramer cyclic voltammetry results, it was unleashed that the aniline tetramer showed 2 redox peaks because of hindrance effect in transitional redox states^39^, however, aniline pentamer exhibited three redox state (Fig. 3A) which can shuttle among all transitional states; therefore, ionic transfer can occur so easily in aniline pentamer than aniline tetramer; moreover, chain length can be effective in ionic transfer, that, aniline pentamer is longer than tetramer one, hence, ions has more space for motion. Therefore, ions mobility is better in aniline pentamer. In alginate-aniline tetramer hydrogel, agarose resulted in gel formation and mechanical feature enhancement; hence, alginate- aniline tetramer units were besieged by agarose network and connection among the aniline oligomer units was hindered and agarose acted as a barricade against ion mobility (Fig. 10). Ascending this system and eliminating this obstruction, agarose chains were participated in conductivity mechanism by coupling the aniline pentamer on agarose chains; hence it was observed that the ionic conductivity of agarose-aniline pentamer was higher than alginate-aniline tetramer; moreover, the low level of activation energy was needed.

Aniline Pentamer is the synthetic oligomer which has the higher thermal stability than natural polymers such as agarose. As it is depicted in Fig. 2E, the Am-Ag-AP has the higher melting point than Ag because of AP melting point. The more AP was added; the higher melting point was occurred. AP has the hydrophobic attributes, whereas agarose has the hydrophilic nature in result by increasing the AP amount in samples, AP tends to self-assemble as micro and nanosphere shape in aqueous solutions, spontaneously. Figure 5 in which the morphology of casted samples was illustrated, exhibits the sphere formation. This behavior may be the cause of the intrinsic viscosity decrement. This phenomenon was observed in some researches that synthetic scaffold had an amphiphilic structure^44,50–52^. Agarose is well-known as a biocompatible and biodegradable polymer which has the non-immunogenic properties; nonetheless, its cellular attachment is low due to the inert structure and the cells form as a sphere on the agarose surface and cannot expand themselves^41,53^; agarose modification by amination process and coupling with AP resulted in amine and carboxyl group formation on the scaffold surface, therefore, it exhibited suitable cellular attachment; Accordance to the Fig. 9A the cellular morphology tends to the spindle morphology and extended on surface with proper neurite out growth. It was surprisingly noticed that the Am-Ag-AP#3 showed the best biocompatibility. This behavior could be related to the biocompatibility of agarose and AP conductivity which cause of better cell proliferation. AP has the conductivity and toxicity, conductivity stimulates cells to have proliferation, adherence and cellular activity; on the other hand, toxicity cause to cell necrosis; As such, there is an optimum percent of AP usage.

Pristine agarose exhibited the higher swelling capacity in comparison with Am-Ag-AP because of its higher affinity toward water than Am-Ag-AP. Aniline pentamer presence in Am-Ag-AP resulted in water uptake decrement because of the hydrophobic nature of aniline pentamer repulsing the water molecule resulted in lower swelling capacity. Conductive scaffolds can be used as a drug delivery system with redox-responsive properties which is safer than other methods because the only voltage stimulation is used and other chemical redox agents are eliminated thus its side effect is decreased. Aniline oligomer exhibited the same electroactivity like polyaniline thus it can be used as a redox-responsive drug release system. Dexamethasone is an anti-inflammatory hydrophobic drug with effective dosage between 10^−4^ M–10^−6^M. Passive drug release depends on substrate and drug hydrophobicity, morphology, porosity^54^. Pure agarose showed the higher release than Ag-AP according to the swelling ratio experiment in which the agarose showed higher SR. Higher SR results in drug molecules mobility enhancement due to the matrix volume and diffusion increment. In aqueous solutions, due to APs tendency to form vesicles which can entrap the Dexamethasone and create the non-covalent interaction between drug and AP, in conjunction with, hydrophobic nature of AP restricts fluid permeation thus initial burst and release rate was lessened. Pure agarose was intact with electrical stimulation and there was no significant difference with passive one, however, electroactive sample was sensitive and exhibited voltage-responsive drug release, voltage applying caused to increment in drug release amount due to variation in APs oxidation/reduction state. This variation cause to morphology alterations or bond cleavages in Am-Ag-AP, hence, the drug release amount was enhanced^55^.

Scaffolds used in tissue engineering must be a biocompatible one to prevent of imposing side effects on tissues. Agarose has no cytotoxicity and it is a suitable material for cells; on the other hand, pure AP showed cytotoxicity and affected adversely in higher concentration. Am-Ag-AP exhibited good biocompatibility with cells because the toxicity of AP was diminished by agarose. The results indicated that the Ag-AP#3 has a good biocompatibility; Fig. 9B and C exhibits the cells behavior under AP and Ag-Ap#3 secreted liquids with 50 mg/ml concentration. PC12 cells were used in nerve regeneration as a model cell which is simply formed as a neuron-like cell. Figure 9A in which the cell attachment of PC12 was shown illustrates that the cells had good adhesion to the scaffold proving the cell biocompatibility with it. Moreover, the neurite length (shown by arrow in Fig. 9A) of PC12 was around 10–20 μm that declared the neurite outgrowth and elongation results in utilizing the bio conductive scaffold without any electrical fields, growth factors and cells such as Schwann cells. Zhao *et al*. showed that the conductivity of scaffold was more important than scaffold modulus^18^. Accordingly, it is supposed that the Am-Ag-AP scaffold would promote axonal growth thus it may be a suitable material for nerve regeneration usage like tubular nerve conduits.

In future works, the mechanical properties of this platform will be optimized and the gene expression and immunocytochemistry will be evaluated. Finally, fabricated scaffold will be implanted in rat as a tubular nerve conduit.

## Conclusion

The novel electro active polymer based on agarose and aniline pentamer has been presented in this study which can be utilized in tissue engineering as a conductive scaffold for neural/cardiac tissues and neural electrodes. Aminated agarose, aniline pentamer and Am-Ag-AP were synthesized and characterized. Conductivity and electroactivity of samples were investigated showing conductivity about 10^−5^ S/cm and 3 oxidation peak also UV spectrum revealed that the AP had 2 transition peaks. Ionic conductivity was increased with temperature enhancing due to the ionic mobility enhancement; in high temperature, activation energy was decreased, hence, ions can transfer so easily. Pure agarose shows the melting peak around 90 °C; however, AP coupling results samples had a more thermal stability and the melting point was increased. Electrochemical impedance spectroscopy was done. The Nyquist plot was used to gain equivalent circuit model based on the neural model which was close to neural cell capacitance. It was inferred that intimacy of scaffold mechanical and conductivity properties to tissue caused better cell growth in addition electrochemical features similarity to cell had a synergic effect on cell proliferation and growth. Agarose-aniline pentamer exhibited lower swelling ratio than pristine agarose due to the hydrophobic nature of aniline pentamer which it repulsed the water molecule and hindered the swelling. Redox-responsive drug release is a safe and multifunctional method (release pattern can be controlled by voltage, ampere alteration and by pulse patterns) therefor the architecting of desire amount of drug release with proper pattern is accessible. Am-AG-AP#3 had the best cell viability and suitable for cell culture. Cells due to the neutral surface of agarose have slight attachment and exhibit spherical morphology, but Am-AG-AP due to the amine and carboxyl group presence showed the proper cell adhesion and neurite out growth. The SEM pictures showed the cell proliferation and cell adhesion to the synthesized polymer.

## Materials and Methods

### Materials

Agarose low electrophoreses, Dimethyl sulfoxide (DMSO), epichlorohydrin and dimethyl formamide (DMF), N-hydroxysuccinimide (NHS) and N, N-di cyclohexyl carbodiimide (DCC) were purchased from Merck. N-Phenyl-p-phenylenediamine, p-Phenylenediamine was received from Sigma-Aldrich.

### NHS-capped Aniline Pentamer Synthesis

NHS-Capped Aniline Pentamer was synthesized in the same route with references. Briefly, the carboxyl/carboxyl capped aniline pentamer was synthesized by reacting of N-Phenyl-p-phenylenediamine and succinic anhydride to produce phenyl/carboxyl capped aniline oligomer with 2 monomers, after that the product was reacted with p-Phenylenediamine to reach aniline pentamer carboxyl/carboxyl capped^56^. In the next procedure, the product was reacted with N-hydroxysuccinimide (NHS) and N,N-di cyclohexyl carbodiimide (DCC) to achieve NHS-Capped Aniline Penamer^44^.

### Aminated Agarose Synthesis

Agarose was dispersed in water, after that by adding sodium hydroxide and epichlorohydrin, the epoxy-activated agarose was obtained. In the next step, the concentrated ammonia was added to reach aminated agarose^57^.

### Aniline Pentamer-Grafted Agarose Synthesis

A 0.5 gr aminated-agarose (Am-Ag) was dissolved in 20 ml hot water, then 10 ml DMSO was added dropwise to the solution. A 0.15 gr NHS-capped aniline pentamer dissolved in 20 ml DMF was dropped to the mixture. The mixture remained in chemical reaction at 50 °C for 24 hours under the nitrogen purge. Later, the product was precipitated in cold ether and collected with Buchner funnel and washed with ethanol. The yield of product was about 0.3 gr.

### Scaffold Characterization

Hydrogen Nuclear Magnetic Resonance (HNMR,400 MHZ) spectra were checked for aniline pentamer characterization using Bruker NMR instrument with Deuterated DMSO. Fourier transform infrared spectroscopy (FTIR) spectra were done on Bruker apparatus using the KBr disk. UV-Visible spectra of AP were recorded by UV-spectrometer. The AP content (C) in the sample was determined by dividing of the slope of samples concentration (P) on pure AP slope (P_0_)(C% = P/P_0_ * 100)^45^. 2,4,6-trinitrobenzenesulfonic acid (TNBS) assay was used for amine designation in aminated-agarose (Am-Ag). Briefly, Am-Ag reacted with TNBS dissolved in sodium bicarbonate buffer for 2hrs at 40 °C. Then, HCl (1 M) and SDS (10%) were used to stop the reaction overnight. The absorbance of the dilute solution was measured at 340 nm^58^.

### Electrochemical and Conductivity Measurement

The conductivity of samples was evaluated by measuring their resistances. Samples were prepared in pellet then volt and ampere were applied, the following equation was used to calculate the conductivity: $(\sigma =\frac{1}{R}\frac{d}{S})$, in which, σ, R, d and S are the conductivity, resistance, thickness and area of samples, respectively^59^. Camphor sulfonic acid (CSA) was used as a dopant as the most suitable dopant to use in tissue engineering^29^. The ionic conductivity was measured in various temperatures and activation energy was calculated using $(\sigma =\,\frac{A}{T}\,exp\,(\frac{{E}_{a}}{RT}))$ in which σ, T, A, R and E_a_ are ionic conductivity, temperature, pre-exponential factor, molar gas constant and activation energy, respectively^39^.

Micro auto lab type ш apparatus was used for cyclic voltammetry (CV) and electrochemical impedance spectroscopy (EIS). Carbon pasted electrode (CPE) modified with 20% polymer was the working electrode, the counter electrode was platinum and the reference electrode was Ag/AgCl. All of which were put in the 1 M HCl solution and the 50 mVs^−1^ was applied. Also, EIS was performed in 0.01 Hz.

### Intrinsic Viscosity Measurement

Ubbelohde viscometer was used to measure the intrinsic viscosity of samples $(\frac{{t}}{{{t}}_{{\rm{0}}}}=\frac{{\rm{\eta }}}{{{\rm{\eta }}}_{0}}=[{\rm{\eta }}])$

t is the time of mixture solution that passes the viscometer. t_0_ is the time that the pure solvent needs to pass the viscometer. ƞ and ƞ_0_ are the mixture solution and solvent viscosity, respectively. Sample properties was reported in Table 1.

**Table 1:** Description and specifications of the samples.

**Sample**	**Aniline percentage in reaction**	**Aniline percentage in sample**	**Conductivity (S/CM)**	**Intrinsic viscosity [ƞ]**
AM-AG-AP#1	10	0.96	9.34 * 10^−6^	2.05
AM-AG-AP#2	20	2.22	2.10 * 10^−5^	2.3
AM-AG-AP#3	30	3.43	5.47 * 10^−5^	2.68
AM-AG-AP#4	40	3.84	8.06 * 10^−5^	2.1
AM-AG-AP#5	50	5.1	1.50 * 10^−4^	1.98

AM-AG-AP: aminated agarose-aniline pentamer.

### Differential Scanning Calorimetry (DSC)

DSC measurement took place in nitrogen atmosphere. Heating rate was 10 °C/min which is swept from 0 to 250 °C and 250 to 0 °C for heating and cooling, respectively.

### Swelling Behavior Evaluation

Dried agarose-aniline pentamer and agarose were weighted and then submerged in distilled water (pH = 7.2 to absorb the water and swell) and after wiping out the superficial water samples were weighted in various time intervals. The swelling ratio was calculated using (SR = (W_s_ − W_d_)/W_d_ × 100) in which SR, W_s_, W_d_ are swelling ratio, swollen weight of sample and dry weight of sample.

### Drug loading and Drug Release Evaluation

Dexamethasone (0.5 g) was dissolved in 50 ml methanol, and 0.25 g dry sample was dipped in methanol congaing drug solution and agitated mildly for 24 h. After that, the sample was washed gently with methanol for removal of the surface bond drug. To calculate the drug loading efficiency, the final drug concentration in the solution was divided into initial drug concentration measured by UV-Vis spectrum. The loading efficiency was then calculated to be around 61.03 ± 4.2%. In order to releasing profile determination, drug loaded sample was submerged in phosphate buffered saline (PBS). At specific interval times, PBS was collected and replaced with a fresh one. The amount of Dexamethasone release was recorded in 242 nm using UV-Visible instrument. For stimulated release, firstly electrodes were put on sample in PBS solution then the desired voltage (0.5) was applied and after that the release amount was recorded. In order to transmute the absorbance data to the drug concentration, the standard curve of dexamethasone was used.

### Biocompatibility Analysis

Cell Proliferation was evaluated using 3-(4,5-dimethylthiazol-2-yl)−2,5-diphenyltetrazolium bromide (MTT) assay. After punching and sterilizing the samples with UV and ethanol for cell survey, they had been immersed in phosphate buffer solution (PBS) for 24 hrs. Samples were put in 24 well plates; the pheochromocytoma (PC12) cells in Dulbecco’s modified eagle medium(DMEM) with 10% fetal bovine serum (FBS) and 10^5^/L penicillin were seeded on the samples, then incubated at 37 °C in 95% moisture and 5% CO_2_. At first, third, fifth and seventh days, the culture media were removed and samples were washed with PBS; then, the MTT was poured on the samples and cells converted the yellow tetrazole to purple formazan. The produced formazan was solved with hydrochloric acid/isopropanol solution (1/50, v/v) after which the amount of absorbance was recorded.

In order to rating the cytotoxicity, samples were incubated at the same condition mentioned above for 24 hours to reach their secreted liquid. 96-well plate seeded PC12 cells were incubated with different concentrations of sample’s extracted liquid (1, 5, 10, 20, 50 mg/ml) in the culture medium. After 24 hours, 100 μL MTT solutions in PBS (5 mg/ml) were added to each well, and then DMSO was poured to solve the produced formazan. The absorbance was recorded at 450 nm.

### Data Availability

All data generated or analyzed during this study are included in this published article (and its Supplementary Information files).
